# Supplementary material for: A scalable platform for efficient CRISPR-Cas9 chemical-genetic screens of DNA damage-inducing compounds
Source: Sci Rep. 2024 Jan 30;14:2508. doi: 10.1038/s41598-024-51735-y (PMC10828508; doi:10.1038/s41598-024-51735-y)
Supplement: Supplementary file 1 — Supplementary Information. [file 41598_2024_51735_MOESM1_ESM.docx]

Supplementary Materials for

**A scalable platform for efficient CRISPR-Cas9 chemical-genetic screens of DNA damage-inducing compounds**

Kevin Lin *et al.*

*Corresponding authors. Email [chadm@umn.edu](mailto:chadm@umn.edu) (C.L.M.) and [azu3jn@virginia.edu](mailto:azu3jn@virginia.edu) (A.K.B.)

**This PDF file includes:**

Figs. S1 to S10

Titles for Supplementary Table 1 to 7

**Fig. S1 Effect of dosage on compound screen hits.**

**
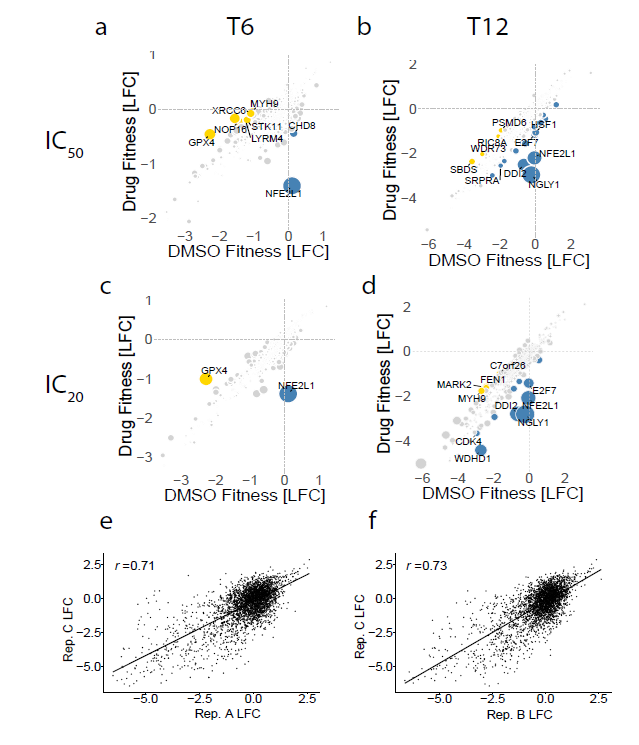
**

**a** Scatterplot of control (DMSO) fitness vs. compound fitness (log_2_ fold change) for T6 BTZ screen at IC_50_ dosage. Negative and positive chemical-genetic interactions (CGIs) are indicated in blue and yellow, respectively. Each point represents a gene. The top 5 negative/positive hits are labeled. **b** Scatterplot of DMSO fitness vs. T12 BTZ fitness (IC_50_). **c** Scatterplot of DMSO fitness vs. T6 BTZ fitness (IC_20_). **d** Scatterplot of DMSO fitness vs. T12 BTZ fitness (IC_20_). **e** Scatter plot of log_2_ fold change (LFC) values, or cell fitness, between technical replicates B and C of the camptothecin (CPT) T12 screen. Pearson’s correlation coefficient (*r*) is reported here. Each point represents one sgRNA. **f** Scatter plot of LFC values between technical replicates A and C of the CPT T12 screen.

**Fig. S2 Correlation matrix for CGI scores.**


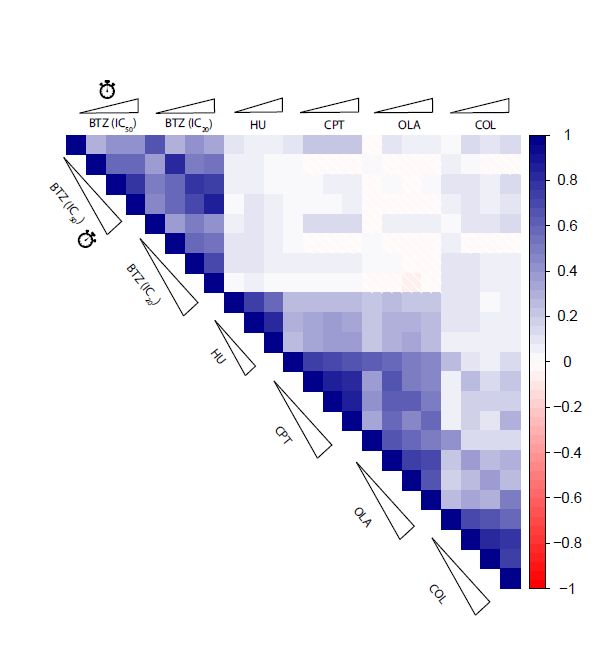


Pearson’s correlation coefficient matrix on CGI scores for each screen. Positive correlations are represented in shades of blue; negative correlations are represented in shades of red. Sliding ramp represents increasing time points.

**Fig. S3 Volcano plots for all compound screens.**


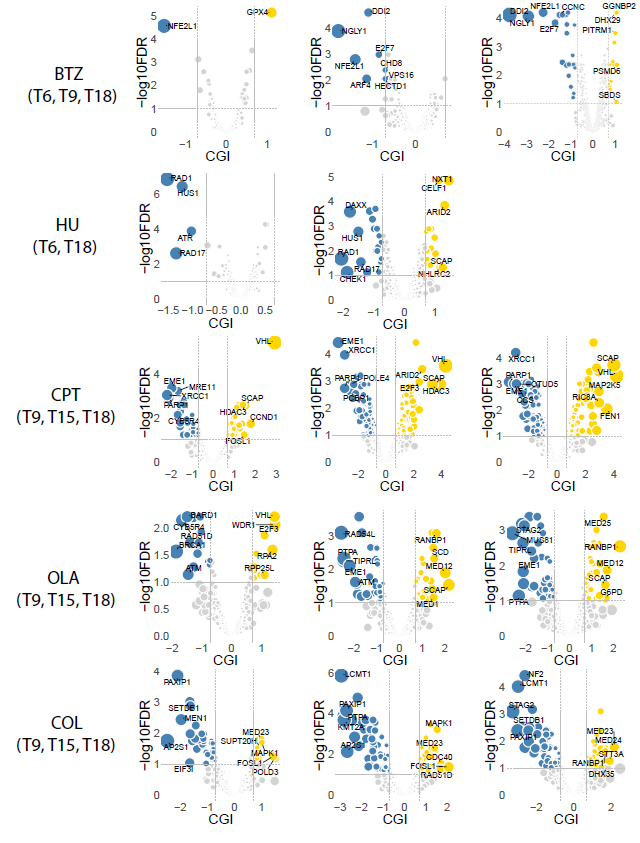


Volcano plot for each compound screen (BTZ = bortezomib, HU = hydroxyurea, CPT = camptothecin, OLA = olaparib, COL = colchicine). Negative and positive CGIs are indicated in blue and yellow, respectively. Each point represents a gene. False discovery rate (FDR) values were estimated using the Benjamini-Hochberg method. Cutoffs for significant CGIs (hits) were set at FDR = 0.1 and |CGI| > 0.7 (gray dashed lines). The top five negative and positive hits are labeled.

**Fig. S4 LFC scatterplots for all compound screens.**


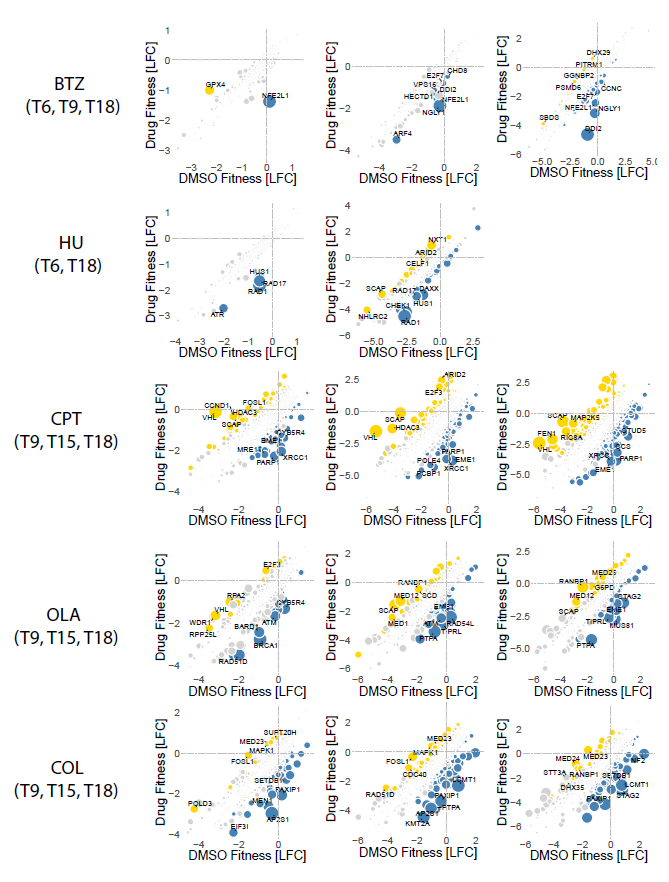


Scatterplot of control (DMSO) fitness vs. compound fitness (BTZ = bortezomib, HU = hydroxyurea, CPT = camptothecin, OLA = olaparib, COL = colchicine). Negative and positive CGIs are indicated in blue and yellow, respectively. Each point represents a gene. The top five negative and positive hits are labeled.

**Fig. S5 Essential gene analysis plots for all essential gene - compound pairs.**


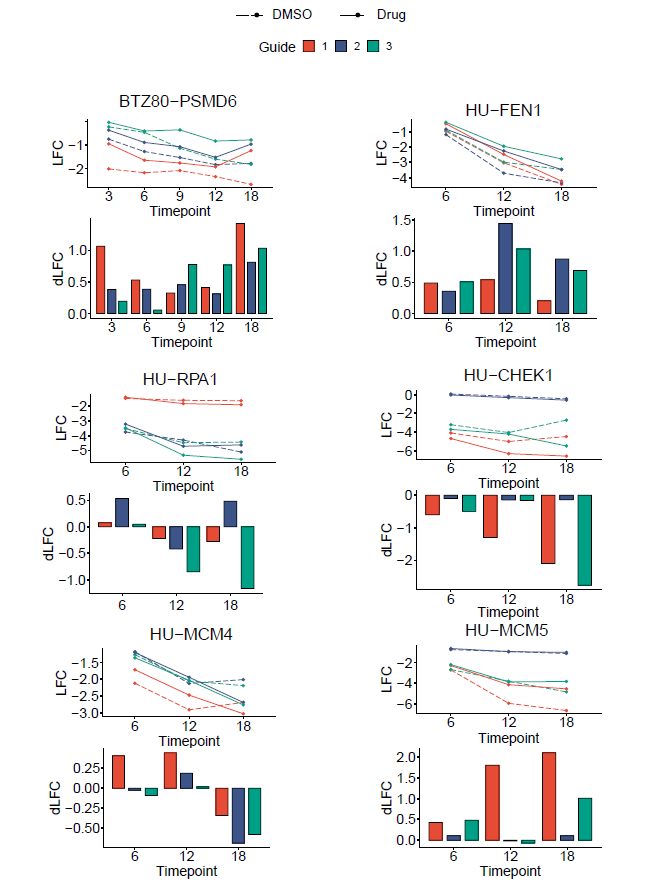


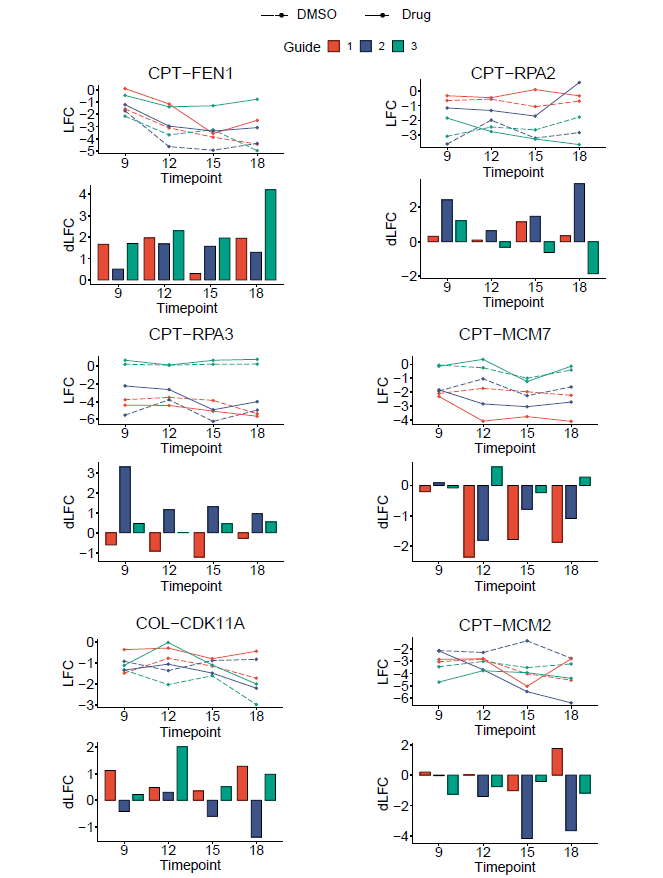


*Top*: Guide-level LFC line plots across time points for each compound-essential gene pair. Orange: sgRNA 1, blue: sgRNA 2, green: sgRNA 3. Dotted line: DMSO; solid line: compound. *Bottom*: Barplot of raw differential LFC (dLFC) score for each guide across time points.

**Fig. S6 Random distribution of total significant hits recovered from simulation of compressed gene library.**


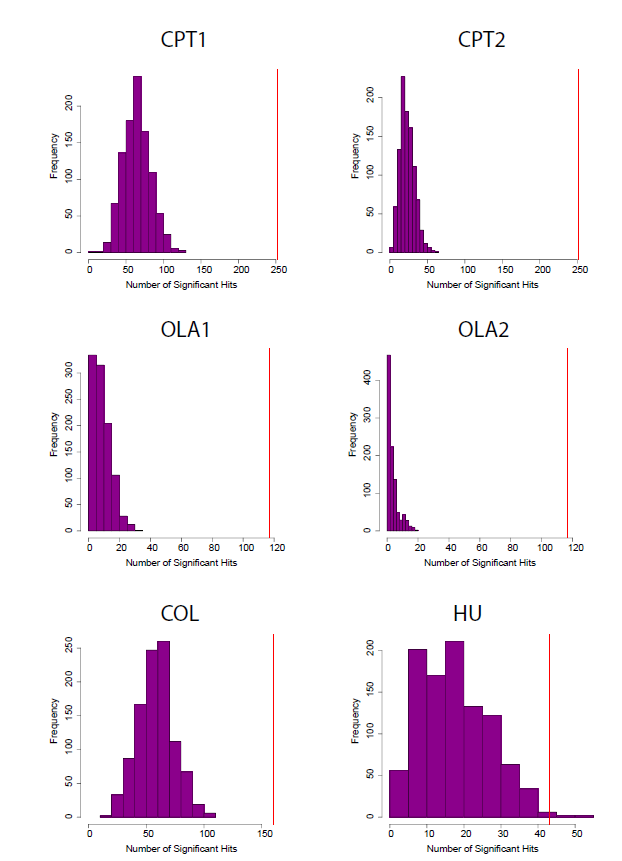


1,000 simulations of hit rate for randomly selected 1,011-gene libraries based on subsets of the genome-wide library. A separate distribution was generated for each compound. Red line: the observed number of significant hits from each respective scalable screen. Genome-wide screen data generated for this study is denoted with “1” following compound name in title. Genome-wide screen data generated by *Olivieri et al.* is denoted with “2” following compound name in title.

**Fig. S7 Barplots of number of hits per compound screen**.


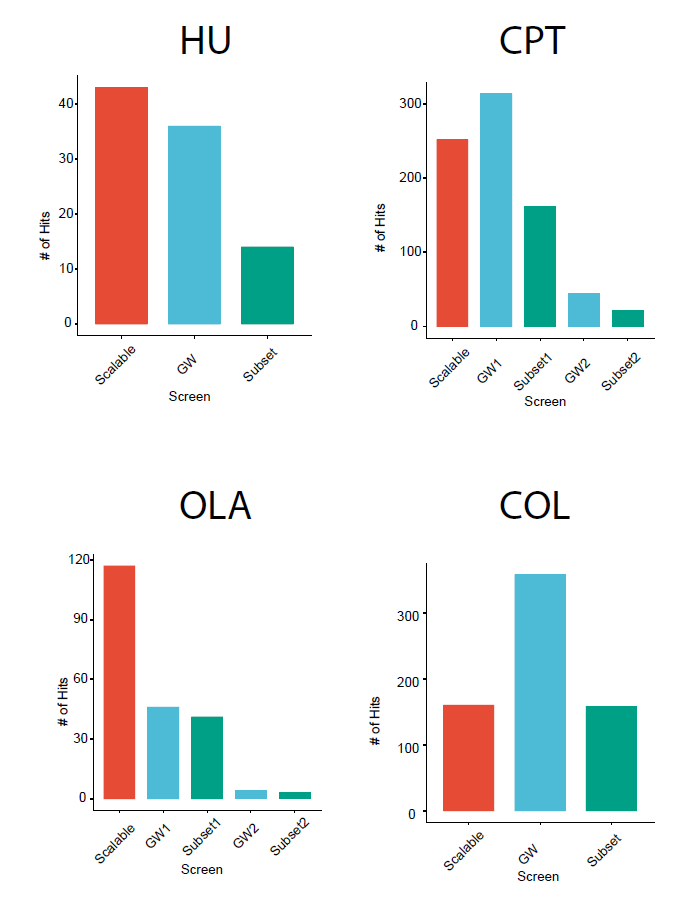


Red: scalable screen. Blue: Genome-wide screen. Green: Genome-wide screen restricted to genes in targeted library (1,011 genes). “1” denotes genome-wide screen performed for this study. “2” denotes genome-wide screen from *Olivieri et al.*

**Fig. S8 SNR plots for all compound screens.**


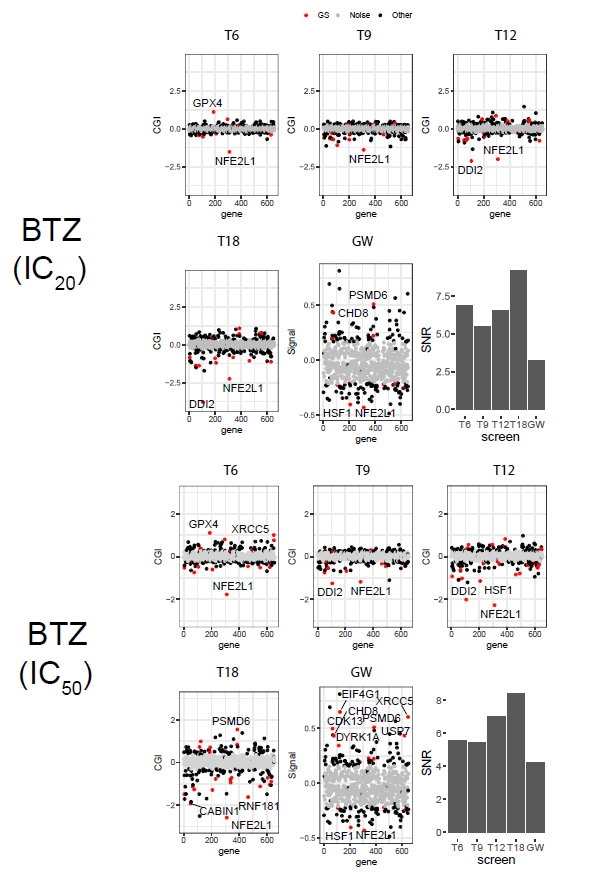


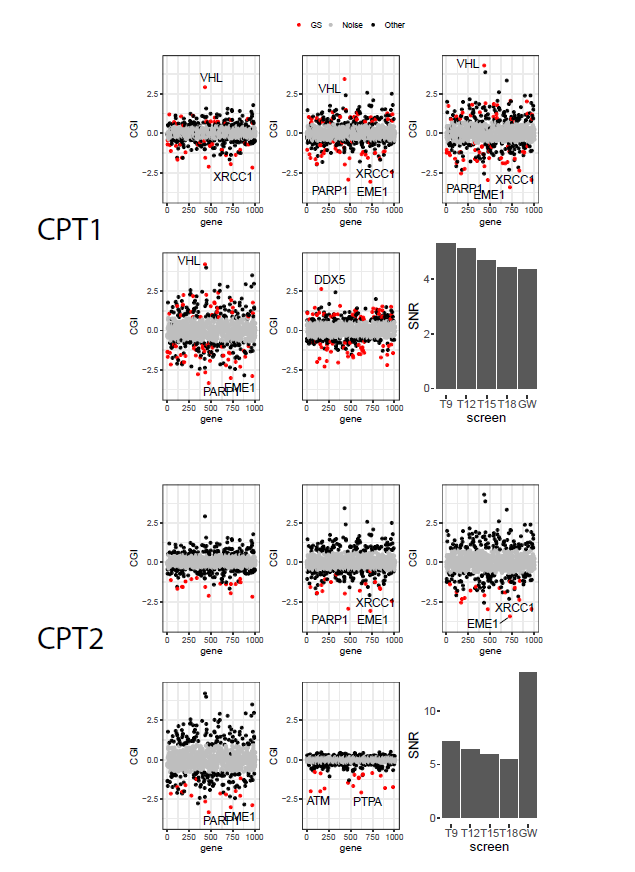


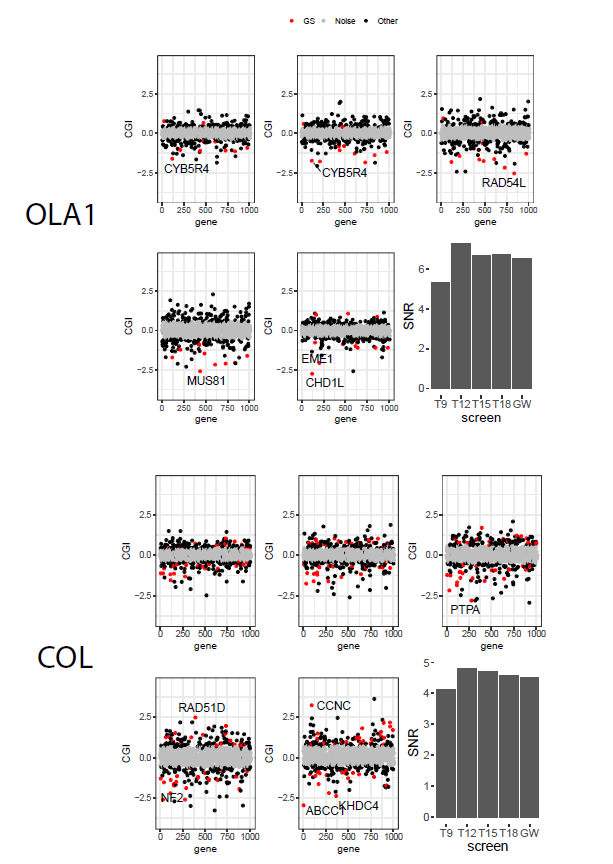


Signal-to-noise ratio (SNR) dotplots. Genes are arranged in alphabetical order from left to right (x-axis), plotted against CGI score (y-axis). Points are divided into 3 categories: 1) gold standard (red dots), 2) background noise (gray dots), and 3) all other genes (black dots). **a** SNR dotplot for bortezomib (at IC_20_ dose). **b** SNR dotplot for bortezomib (at IC_50_ dose). **c** SNR dotplot for camptothecin (vs. our genome-wide screen). **d** SNR dotplot for camptothecin (vs. genome-wide screen from *Olivieri et al.*) **e** SNR dotplot for olaparib (vs. genome-wide screen conducted for this study). Note that comparison vs. genome-wide screen from *Olivieri et al.* was not included because there were not enough hits from the genome-wide screen to conduct SNR analysis. **f** SNR dotplot for colchicine (vs. genome-wide screen conducted for this study).

**Fig. S9 CGI heatmap for all other curated pathways.**


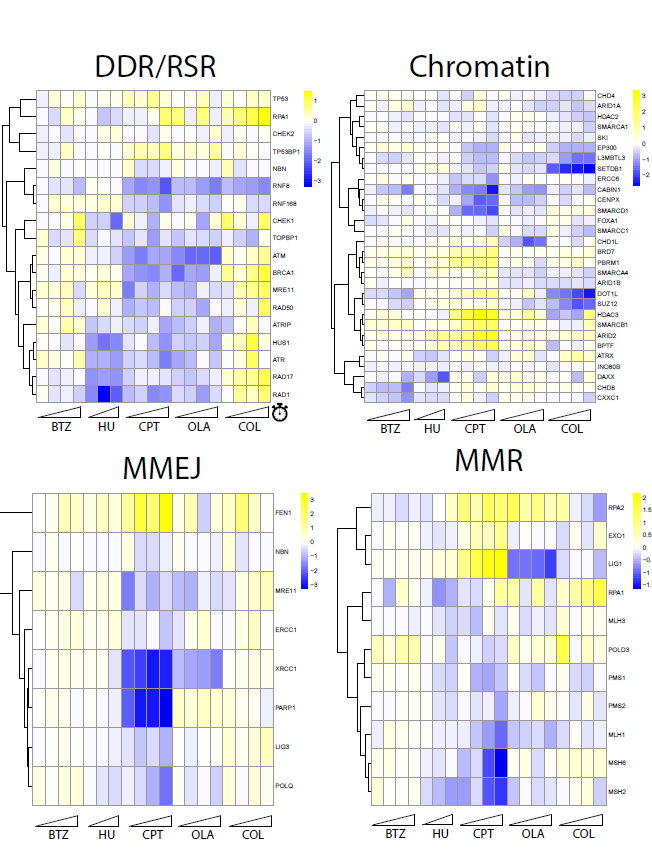


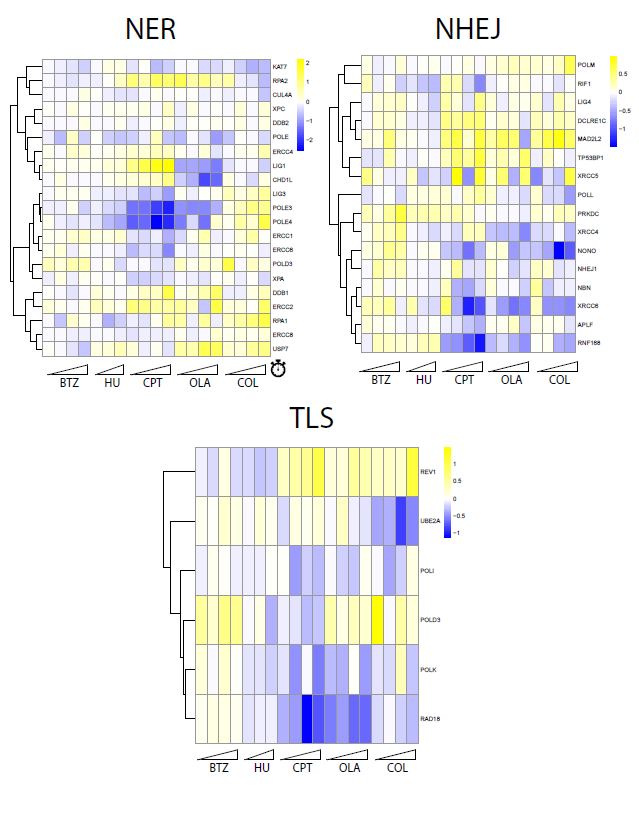


**a** Heatmap of CGI scores for DNA damage response / replication stress response (DDR/RSR) pathway genes, using average-linkage hierarchical clustering. Blue represents negative CGI score, yellow represents positive CGI score, white represents zero CGI score. X-axis ordered by screen time point (sliding ramps). **b** Heatmap for chromatin remodeling genes. **c** Heatmap for microhomology-mediated end joining (MMEJ) pathway. **d** Heatmap for mismatch repair (MMR) pathway. **e** Heatmap for nucleotide excision repair (NER) pathway. **f** Heatmap for non-homologous end joining (NHEJ) pathway. **h** Heatmap for translesion synthesis (TLS) genes.

**Fig. S10 Effect of CGI cutoffs on library hit rate.**

**
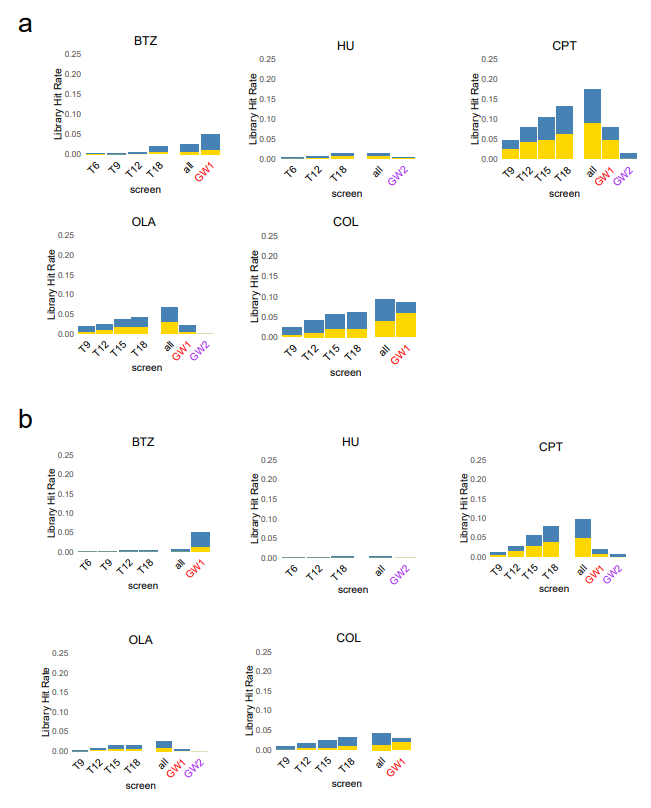
**

**a** Barplots of library hit rate per screen, with hits defined as |CGI| > 1 and FDR < 10%. Blue represents negative CGI hits, yellow represents positive CGI hits. For each compound, a genome-wide screen was selected for comparison (see Table 1). Red label: genome-wide screen. GW1: genome-wide screen performed for this study. GW2: genome-wide screen from Olivieri *et al.* All: union of hits across all time points for a given screen. **b** Barplots of library hit rate per screen, with hits defined as |CGI| > 1.5 and FDR < 10%.

**Separate Files**

**Supplementary Table 1:** Significant hits list for each scalable compound screen

**Supplementary Table 2:** Number of hits and overlapping hits for each screen

**Supplementary Table 3:** GO:Biological Process enrichment for each screen

**Supplementary Table 3:** GO:Biological Process enrichment on hits unique to scalable screens

**Supplementary Table 5:** Categorization of targeted library genes and guide sequences

**Supplementary Table 6:** Manual curation of 11 DDR-related pathways

**Supplementary Table 7:** GO enrichment across varying CGI cutoffs. GO enrichment analysis was completed at three different cutoffs (|CGI| > 0.7, FDR < 10%; |CGI| > 1, FDR < 10%; |CGI| > 1.5, FDR < 10%). The union of hits across all time points for each screen was evaluated for enrichment. Enrichment statistics are provided for all GO terms with non-zero overlap with >10 and <300 total annotations.
